# AI-Assisted Edema Map Optimization Improves Infarction Detection in Twin-Spiral Dual-Energy CT

**DOI:** 10.3390/brainsci15080821

**Published:** 2025-07-31

**Authors:** Ludwig Singer, Daniel Heinze, Tim Alexius Möhle, Alexander Sekita, Angelika Mennecke, Stefan Lang, Stefan T. Gerner, Stefan Schwab, Arnd Dörfler, Manuel Alexander Schmidt

**Affiliations:** 1 Institute of Neuroradiology, University Hospital Erlangen, Friedrich-Alexander-University Erlangen-Nürnberg, 91054 Erlangen, Germany; ludwig.singer@uk-erlangen.de (L.S.); daniel.heinze@uk-erlangen.de (D.H.); tim.moehle@fau.de (T.A.M.); angelika.mennecke@uk-erlangen.de (A.M.); s.lang@uk-erlangen.de (S.L.); arnd.doerfler@uk-erlangen.de (A.D.); 2 Department of Neurology, University Hospital Erlangen, Friedrich-Alexander-University Erlangen-Nürnberg, 91054 Erlangen, Germany; alexander.sekita@uk-erlangen.de (A.S.); stefan.gerner@uk-erlangen.de (S.T.G.); stefan.schwab@uk-erlangen.de (S.S.); 3 Department of Neuroradiology and Neurovascular Therapy, Klinikum Main-Spessart, 97816 Lohr am Main, Germany

**Keywords:** stroke, Dual-Energy, endovascular stroke therapy, edema map, AI-assisted post-processing

## Abstract

Objective: This study aimed to evaluate whether modifying the post-processing algorithm of Twin-Spiral Dual-Energy computed tomography (DECT) improves infarct detection compared to conventional Dual-Energy CT (DECT) and Single-Energy CT (SECT) following endovascular therapy (EVT) for large vessel occlusion (LVO). Methods: We retrospectively analyzed 52 patients who underwent Twin-Spiral DECT after endovascular stroke therapy. Ten patients were used to generate a device-specific parameter (“y”) using an AI-based neural network (SynthSR). This parameter was integrated into the post-processing algorithm for edema map generation. Quantitative Hounsfield unit (HU) measurements were used to assess density differences in ischemic brain tissue across conventional virtual non-contrast (VNC) images and edema maps. Results: The median HU of infarcted tissue in conventional mixed DECT was 33.73 ± 4.58, compared to 22.96 ± 3.81 in default VNC images. Edema maps with different smoothing filter settings showed values of 14.39 ± 4.96, 14.50 ± 3.75, and 15.05 ± 2.65, respectively. All edema maps demonstrated statistically significant HU differences of infarcted tissue compared to conventional VNC images (p<0.001) while maintaining the density values of non-infarcted brain tissue. Conclusions: Enhancing the post-processing algorithm of conventional virtual non-contrast imaging improves infarct detection compared to standard mixed or virtual non-contrast reconstructions in Dual-Energy CT.

## 1. Introduction

Stroke is a leading cause of morbidity, disability, and mortality worldwide [1]. Endovascular therapy (EVT) is a cornerstone in acute stroke therapy, with recent studies even showing that selected patients in a late time window or with early ischemic changes benefit from this [2,3,4,5]. Typical follow-up procedures after EVT include a post-interventional CT scan, usually within 24 h, to visualize the degree of infarction, exclude intracranial hemorrhage (ICH), and to plan further management of the patient. A frequent finding in these early post-interventional CT scans are intraaxial or subarachnoid hyperdensities occurring due to disruption of the blood–brain barrier or leakage of contrast material into the subarachnoid space [6,7].

Conventional single-energy CT (SECT) acquires images at a single, fixed photon energy, so materials with similar attenuation characteristics—and thus overlapping Hounsfield unit values—appear virtually indistinguishable. Consequently, extravasation of iodinated contrast material into infarcted tissue can be mistaken for hemorrhagic transformation [8,9].

Compared to SECT, Dual-Energy CT (DECT) employs high- and low-energy X-rays to measure different attenuation levels and further characterize these hyperdensities using a three-material decomposition algorithm in different compounds [9,10,11,12]. Current acquisition methods in routine imaging include classical dual source scanning with two X-ray tubes, rapid voltage switching, dual-layer detector, split filter technique, and more recently sequential scanning (Dual- or Twin-Spiral Dual-Energy CT) [13].

Previous studies have demonstrated that DECT performed after mechanical thrombectomy can differentiate hyperdensities seen on the post-interventional CT—distinguishing contrast extravasation from hemorrhage—and improve the detection of infarcted brain tissue compared to conventional SECT [14,15,16].

AI solutions in acute stroke imaging are already employed for a wide range of tasks including automated large vessel occlusion (LVO) detection, hemorrhage detection, and automated evaluation of perfusion imaging on CT or MR [17]. Concurrently, deep learning reconstruction and denoising algorithms (e.g., Deep Resolve) have reduced MRI stroke exams to a total time of under 10 min [18,19,20].

To further refine the post-processing of the Dual-Energy CT, we adapted the method of edema map generation using a device-specific parameter “y” [21,22]. We employed an AI-based neural network to separate gray and white matter and overlay these masks onto the CT scan to measure the respective Hounsfield units (HU) of gray and white matter. From this we calculated the device-specific parameter that suppresses gray-white differentiation. We hypothesize that if the brain appears uniformly gray, it is easier to spot subtle changes in density, ultimately increasing the visibility and identification of infarcted tissue.

## 2. Materials and Methods

### 2.1. Patients

We retrospectively analyzed 52 patients (22 males, 30 females, mean age 70 years, IQR: 61–85) between July 2023 and March 2025 who had a Twin-Spiral DECT scan following EVT after large vessel occlusion (LVO). A total of 11 patients had an occlusion of the ICA, 38 of the MCA (23 M1-segment, 14 M2-segment, 1 M3-segment), and 3 patients had an occlusion in the posterior circulation (1 VA, 2 BA). Inclusion criteria required the availability of DECT imaging within 24 h following EVT; large vessel occlusion (LVO) confirmed on initial stroke CT and successful EVT. There were no exclusion criteria. When accessible, follow-up CT or MRI scans were used as the reference standard to confirm infarction or contrast staining (n=32). In the absence of follow-up imaging, infarction was identified by comparing pre-stroke imaging with NCCT and CT perfusion acquired during the initial stroke workup (n=20). Reasons for absent follow-up imaging included severe strokes with subsequent patient death or minor strokes where patients were discharged home early.

### 2.2. Image Acquisition and Post Processing

A DECT scan was performed in all patients using a single source DECT scanner (X.ceed, Siemens Healthineers, Forchheim, Germany). Parameters of the scan protocol in all patients were tube voltages of 80 kV and tin (Sn) filtered 150 kV. A slice thickness of 1 mm, a pitch factor of 0.55, and a CARE kV Image Quality-setting of 260 were selected. The mean computed tomography dose index volume (CTDIvol) was 44.95 ± 4.59 mGy. Image reconstruction included both virtual non-contrast (VNC) and standard mixed images in axial orientation and an image matrix of 512 × 512 mm.

Post-processing and dataset reconstruction were fully automated on the CT scanner’s workstation (syngo.via, CT Brain Hemorrhage, VA.40 client 4.0, Siemens Healthineers AG, Erlangen, Germany). Virtual monoenergetic reconstructions at 87 keV with slice thicknesses of 3 mm in axial, sagittal, and coronal orientations were generated, representing classical non-contrast CT. Additionally, color-coded iodine overlay images, VNC images at default settings, and edema maps were reconstructed at three different post-processing configurations, labeled as “Resolution” (1, 3, and 5) in the interface. This parameter controls the strength of the spatial smoothing filter applied during image reconstruction, where higher values correspond to stronger smoothing and lower image noise.

### 2.3. Image Conversion

Mixed, low, and high energy datasets of the Dual-Energy CT were converted to nifti format. The mixed energy series was then converted into a synthetic MPRAGE using SynthSR convolutional neural network (CNN) [23]. Synthetic MPRAGE was then segmented into gray and white matter using SPM12. Segmentation files were registered and overlaid onto low- and high-energy CT scans. Voxel-based Hounsfield units of gray and white matter were measured using Python 3.14 [24].

### 2.4. Image and Statistical Analysis

Hounsfield units (HU) were measured by a single experienced evaluator (L.S.) who manually placed one equally sized spherical ROI within the ischemic lesion on the conventional CT image, with all placements reviewed and validated by a supervising neuroradiologist (M.A.S.). The identical ROI location was then applied across all VNC and edema map reconstructions at varying spatial smoothing filter settings and iodine ratios. Data was tested for normal distribution using the Shapiro–Wilk test. Paired *t*-tests were applied to normally distributed data, while Wilcoxon signed-rank tests were used for non-normally distributed data. Statistical analyses were performed using Python 3.14 and R Version 4.5.0 [24,25].

## 3. Results

**Figure 1:**
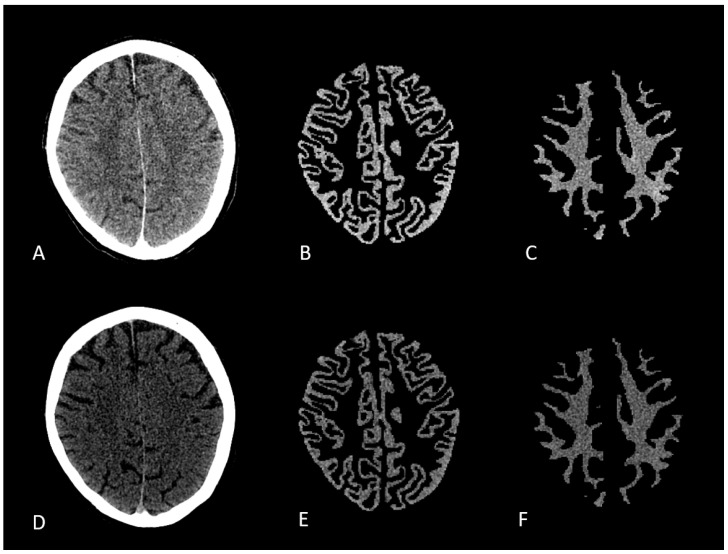
(**A**–**C**): 80 kV CT scan with respective GM and WM segmentation masks; (**D**–**F**): tin-filtered 150 kV scan with respective GM and WM segmentation masks.

Of the 52 patients analyzed, 10 were used to calculate the device-specific parameter “y”: their CT scans were converted into synthetic MPRAGEs and overlaid as described above. We limited this derivation cohort to 10 patients because our voxel-wise analysis included over 28 million voxels and revealed very low variance between regions, indicating that a larger sample was unnecessary for robust parameter estimation (Figure 1). The total number of analyzed voxels was 7,277,096 (GM) and 7,108,959 (WM) in high-energy CT scans, and 7,291,076 (GM) and 7,122,897 (WM) in low-energy scans. The median density of GM in the high-energy scans was 32 HU (SD: 2.19), while WM had a median of 27.95 HU (SD: 1.27). In low-energy scans, GM had a median density of 47.84 HU (SD: 3.25), and WM had 39.92 HU (SD: 2.25). Following measurement of median HU values, we computed the suppression ratio *y* using Mohammed et al.’s formula:

(1) $$y=\left(GM_{\mathrm{low}}-WM_{\mathrm{low}}\right)/\left(GM_{\mathrm{high}}-WM_{\mathrm{high}}\right)$$

Substituting our values yielded the following: y=1.95 [21] (see Table 1).

**Figure 2:**
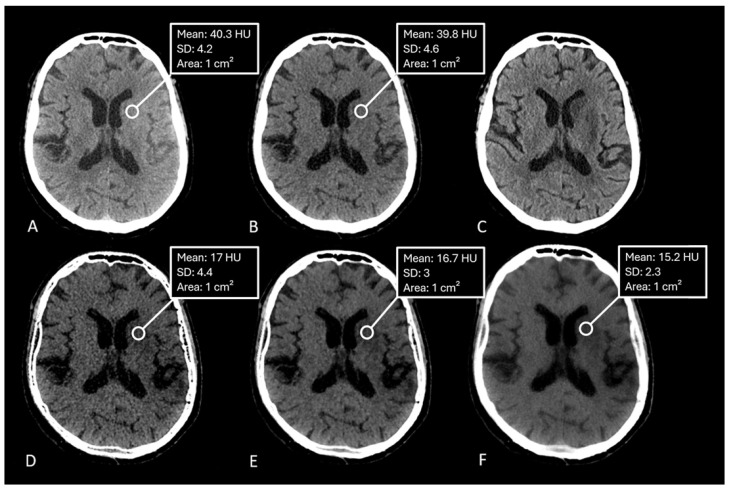
CT-Images of a 71 year old male after recanalization of a M1-occlusion on the left. (**A**): Mixed images with no visible infarction of the basal ganglia, (**B**): conventional virtual non-contrast map with barely visible infarction, (**C**): follow-up CT after 5 days with Infarction of the basal ganglia, (**D**): edema map at smoothing level 1 with visible infarction of the basal ganglia, (**E**): edema map at smoothing level 3 with clearly visible infarction of the basal ganglia and good image quality, (**F**): edema map at smoothing level 5 with clearly visible infarction of the basal ganglia.

**Figure 3:**
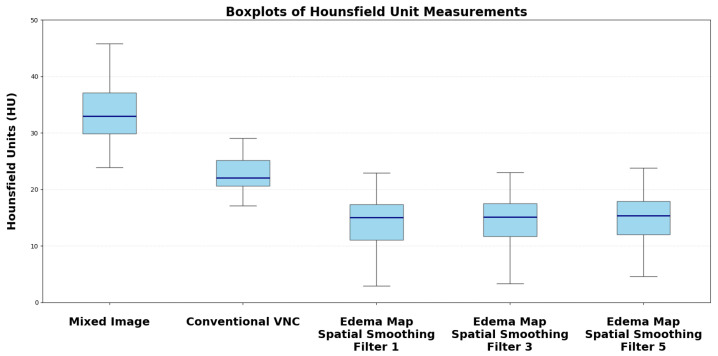
Boxplots of Hounsfield unit distribution of different reconstruction parameters of VNC and edema maps.

**Figure 4:**
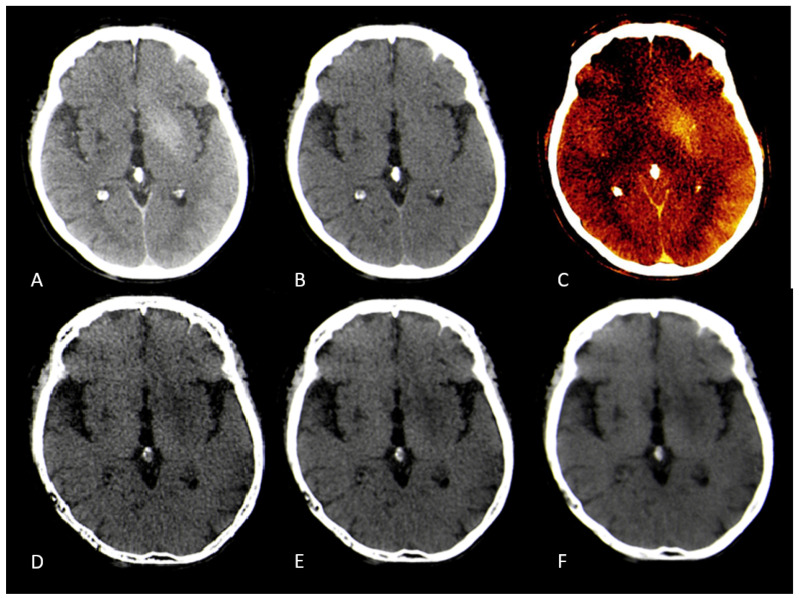
CT images of a 90 year old female after recanalization of a left-sided M1-occlusion. (**A**): Conventional mixed images with contrast staining of basal ganglia, (**B**): conventional virtual non-contrast map with barely visible infarction, (**C**): iodine map confirming contrast staining of basal ganglia, (**D**): edema map at smoothing level 1 with clearly visible infarction of the basal ganglia and high image noise, (**E**): edema map at smoothing level 3 with clearly visible infarction of the basal ganglia and good image quality, (**F**): edema map at smoothing level 5 with clearly visible infarction of the basal ganglia and loss of detail due to high smoothing.

The median measured Hounsfield unit (HU) of the infarction area on conventional mixed CT images was 33.73 HU (SD: 4.58), for the default VNC-map 22.96 HU (SD: 3.81), for edema maps at smoothing level 1 (14.39 HU, SD: 4.98), smoothing level 3 (14.50 HU, SD: 3.75), and smoothing level 5 (15.05 HU, SD: 2.65) (Figure 2). Normality testing using the Shapiro–Wilk test indicated that median HU values of default VNC and of edema maps at level 1 of the smoothing filter followed a normal distribution, while all other parameters were non-normally distributed. Significant differences in median Hounsfield unit values were observed between conventional VNC images and edema maps at all levels of smoothing (p<0.001) (Table 2, Figure 3 and Figure 4).

## 4. Discussion

Routine imaging after endovascular stroke therapy (EVT) usually includes CT or MRI imaging to determine the extent of infarction or to identify hemorrhagic transformation which occurs in approximately 15% of patients [26,27,28]. Common findings on the post-interventional CT are intraparenchymal or subarachnoid hyperdensities which could resemble hemorrhage or contrast extravasation [29]. Dual-Energy CT has already been shown to reliably differentiate between hemorrhage or contrast extravasation using virtual non-contrast and iodine maps [15,27,30]. Several previous studies have also shown that virtual non-contrast images are superior in identifying infarction tissue compared to conventional single energy CT [12,21,22,31].

In our study we adjusted the post-processing parameters of Twin-Spiral Dual-Energy CT by implementing a device-specific parameter “y” for edema map generation analogous to Mohammed et al.’s study [21]. Compared to previous studies we derived this parameter from a voxel-wise measurement of gray and white matter densities using AI-assisted brain segmentation. We hypothesize that by suppressing the gray–white matter contrast, the brain parenchyma appears homogenous facilitating the detection of subtle hypodensities, including cytotoxic edema [21].

Our results confirm this hypothesis. Edema maps at varying strengths of the smoothing filter show significantly lower Hounsfield unit (HU) values in infarction tissue compared to conventional VNC images (all p<0.001). Using the device-specific parameter “y” at a smoothing level of 1 resulted in the largest absolute reduction of infarct HU values. In these settings, the image was very noisy compared to mixed CT or the conventional VNC map. With an increase of the spatial smoothing filter to a setting of 3 there was a marked increase of image quality while maintaining the same infarction detection compared to a smoothing setting of 1 (p=0.10) (Figure 3).

Compared to previous analyses that used manual ROI-based HU measurements to calculate post processing settings, we performed a voxel-based analysis with more than 28 million voxels analyzed, providing a more robust data foundation [21,22,31]. Additionally, other analyses focused on already established acquisition methods of the DECT like dual source or dual layer scanning. Twin-Spiral Dual-Energy CT is a relatively recent method in spectral CT acquisition relying on sequential kV switching rather than conventional dual-source or dual-layer configurations. Through this difference it may produce different spectral information compared to the already established methods making it essential to validate post-processing strategies such as edema map generation. A possible drawback of this type of acquisition—due to the use of double-spiral sequences—is the prolonged acquisition time, which may render the examination susceptible to motion artifacts. However, in all our patients, this was not the case and no disturbances of image quality were observed.

The clinical implications of our edema maps include a more accurate and earlier visualization of the full extent of infarction and differentiation of hemorrhagic transformation from contrast staining. This may reduce the need for additional imaging studies, shorten the interval to therapeutic decisions—such as safe initiation of anticoagulation or antithrombotic agents, particularly in atrial fibrillation to prevent recurrent embolic stroke—and improve patient stratification for neuroprotective or decompressive interventions [32,33].

Several limitations of our study have to be acknowledged. The relatively small sample size and retrospective nature of the analysis are well known to be subject to various biases. Furthermore, while the use of synthetic T1-MPRAGE images generated from CT data enabled gray and white matter segmentation; there is a risk of image distortion and that the segmentations may not fully resemble true anatomical gray and white matter distributions. Additionally, MRI—often considered the gold standard for infarct detection—was not available in the majority of cases for comparison.

Further studies should verify if the device-specific parameter ‘y’ can be generalized to other Dual-Energy capable scanners and acquisition methods. Such validation may facilitate easier infarction detection compared to conventional VNC images. Additionally, there should be a focus on the benefits of Dual-Energy CT in the setting of acute ischemic stroke employing edema maps and virtual monoenergetic reconstructions to identify early ischemic changes [34,35]. In summary, refining the post-processing of conventional virtual non contrast images has the potential to improve infarct detection after EVT compared to conventional SECT and conventional VNC maps.

## 5. Conclusions

In this study, we demonstrate that an AI-assisted device-specific adjustment of post-processing parameters for Twin-Spiral DECT produces edema maps that significantly enhance the visualization of ischemic brain tissue compared to conventional VNC images and SECT.

By leveraging an AI-based suppression parameter “y” and voxel-wise analysis of gray and white matter across >28 million voxels, our method renders the brain uniformly gray, amplifying subtle cytotoxic hypodensities and delineating the full infarct extent while reliably distinguishing contrast staining from hemorrhage.

## Figures and Tables

**Table 1:** Number of analyzed voxels and median HU values (SD) for high-energy and low-energy CT scans.

Scan Type	Number of Analyzed Voxels	Median HU (SD)
High-Energy CT (GM)	7,277,096	32 (2.19)
High-Energy CT (WM)	7,108,959	27.95 (1.27)
Low-Energy CT (GM)	7,291,076	47.84 (3.25)
Low-Energy CT (WM)	7,122,897	39.92 (2.25)

**Table 2:** Median Hounsfield Unit (HU) values with standard deviation (SD) and corresponding *p*-values for comparisons against Siemens default VNC.

Reconstruction	Median HU (SD)	*p*-Value vs. Mixed Image/VNC
Conventional Mixed Image	33.73 (4.58)	
Default VNC	22.96 (3.85)	
Edema Map Resolution 1	14.39 (4.98)	p<0.001
Edema Map Resolution 3	14.50 (3.75)	p<0.001
Edema Map Resolution 5	15.05 (2.65)	p<0.001

## Data Availability

The raw data supporting the conclusions of this article will be made available by the authors on request.

## Abbreviations

The following abbreviations are used in this manuscript:

AI	Artificial Intelligence
CNN	Convolutional Neural Network
CT	Computed Tomography
DECT	Dual-Energy Computed Tomography
DWI	Diffusion-Weighted Imaging
EVT	Endovascular Stroke Therapy
GM	Gray Matter
HU	Hounsfield Unit
ICA	Internal Carotid Artery
ICH	Intracranial Hemorrhage
LVO	Large Vessel Occlusion
MCA	Middle Cerebral Artery
MPRAGE	Magnetization Prepared Rapid Acquisition Gradient Echo
MRI	Magnetic Resonance Imaging
PACS	Picture Archiving and Communication System
ROI	Region of Interest
SECT	Single-Energy Computed Tomography
VNC	Virtual Non-Contrast
WM	White Matter
